# Analysis of the Haematological Phenotype and Molecular Characteristics of Rare Abnormal Haemoglobin

**DOI:** 10.1002/mgg3.70012

**Published:** 2024-09-12

**Authors:** Yanfen Ge, Guansheng Zheng, Luhua Xian, Yanfei Luo, Junru Liu, Ting Lin, Wenhao Cui, Yujing Yang, Huizhuang Shan

**Affiliations:** ^1^ Department of Clinical Laboratory, Guangdong Provincial People's Hospital (Guangdong Academy of Medical Sciences) Southern Medical University Guangzhou Guangdong China; ^2^ KingMed School of Laboratory Medicine Guangzhou Medical University Guangzhou Guangdong China

**Keywords:** haemoglobin, haemoglobinopathy, Hb Fukuyama, Hb Port Phillip, Hb Saint Etienne

## Abstract

**Background:**

Haemoglobinopathy refers to a group of common monogenic inherited conditions associated with variations in the haemoglobin molecule; however, there is relatively limited reporting on abnormal haemoglobinopathy in the Chinese population, especially rare abnormal haemoglobin (Hb). The aim of this study was to explore the clinical characteristics of haemoglobinopathy to supplement data for the epidemiological investigation of Hb variants in Guangdong province of China.

**Methods:**

Peripheral blood was collected from five patients (including a family) for Complete blood count, Hb electrophoresis, High‐performance liquid chromatography analysis and degenerative globin body testing. Hb variants were further analysed by PCR and DNA sequencing.

**Results:**

The research subjects were diagnosed with different types of abnormal Hb. The blood routine of the Hb Fukuyama (HBB:c.232C>T) diagnosed individual showed microcytic hypochromic anaemia, with a lower Hb level (64 g/L), mean corpuscular volume (MCV) of 71.5 fL and mean corpuscular haemoglobin (MCH) of 21.5 pg. Individuals diagnosed with Hb Port Phillip (HBA2:c.275T>C) exhibit a MCH level that is slightly below average, at 26.4 pg. The Hb Saint Etienne (HBB:c.279C>G) diagnosed individual showed macrocytic hypochromic anaemia, and the proband had a low Hb level (116 g/L), MCV of 102.2 fL and MCH of 29.4 pg.

**Conclusion:**

We confirmed the presence of Hb Fukuyama (HBB:c.232C>T) in China for the first time. Three rare patients with the Hb Saint Etienne (HBB:c.279C>G) phenotype and one patient with Hb Port Phillip (HBA2:c.275T>C) phenotype were included. Our research enriches the gene mutation map of haemoglobinopathy in Guangdong province and improves the detection system of haemoglobinopathy for population prevention and eugenics.

## Graphical abstract

The research subjects were diagnosed with different types of abnormal haemoglobin. We confirmed the presence of *Hb Fukuyama* in China for the first time. Three rare patients with the *Hb Saint Etienne* phenotype and one patient with *Hb Port Phillip* phenotype were included.

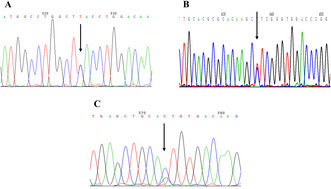

## 1 Introduction

Abnormal haemoglobin refers to haemoglobin with an abnormal globin chain molecular structure, and its primary structural abnormality can occur in any of the α‐, β‐, γ‐ and δ‐globins. To date, 1426 Hb variants have been found worldwide, including 466 α_2_‐globin variants and 953 β‐globin variants (http://globin.bx.psu.edu/cgi‐bin/hbvar/counter). The overall prevalence of abnormal haemoglobin in China is 0.26% (Huang et al. 2019). Haemoglobin structural abnormalities including thalassemia‐like variants, haemolytic/unstable abnormal haemoglobins, abnormal oxygen affinity haemoglobins and other abnormal haemoglobins (Hui et al. 2022). Previous studies reported that the most abnormal haemoglobins do not change their physiological function (Ahmed, Ghatge, and Safo 2020; Drvenica et al. 2022), and only 1/3 of abnormal haemoglobins have a change in physiological function, resulting in clinical symptoms, the most common of which is haemolytic anaemia.

Unstable haemoglobins are one of the causes of congenital non‐spherocytic haemolytic anaemia, and nearly 200 unstable haemoglobins have been identified (De Simone et al. 2022; Giardine et al. 2021). Hundreds of these haemoglobins showed haemolysis or abnormal oxygen affinity, and another 100 had no haematological abnormalities but were unstable at in vitro assays. It is easily deposited on the cell membrane due to instability. The lifespan of the affected red blood cells are shortened and symptoms of haemolysis appear to varying degrees. The clinical manifestations of unstable Hb vary greatly, sometimes manifesting as chronic haemolytic anaemia with paroxysmal exacerbations, usually induced by coinfection or taking oxidative drugs (Williamson 1993).

Understanding abnormal haemoglobin is crucial for the diagnosis and treatment of clinically related diseases. However, research on abnormal haemoglobin is relatively rare in Guangdong Province of China (Xian et al. 2022). In this study, three rare abnormal haemoglobins, Hb Fukuyama (HBB:c.232C>T), Hb Port Phillip (HBA2:c.275T>C) and Hb Saint Etienne (HBB:c.279C>G), were found through Hb electrophoresis and globin gene detection, and their related haematological parameters and clinical features were analysed.

## 2 Materials and Methods

### 2.1 Research Population

The study population was outpatient and inpatient patients who underwent haemoglobin electrophoresis testing at Guangdong Provincial People's Hospital, Guangdong Province, China, between November 2013 and December 2022. Five cases of rare abnormal haemoglobin were included in the study, comprising two male individuals aged 65 (Patient 1) and 31 (Patient 2), respectively. One family included the proband (female), her daughter and her sister (Patients 3–5), with ages 23, 4 and 29, respectively.

### 2.2 Complete Blood Count (CBC)

A Coulter LH750 automatic blood cell analyser from Beckman Company of the United States was used.

### 2.3 Hb Typing by Alkaline Gel Electrophoresis and Capillary Electrophoresis (CE)

The automatic rapid system (SPIFE3000) and its pH 8.6 agar gel reagent were purchased from Helena Company in the United States; the Capillarys 2 flex‐piercing automatic capillary electrophoresis instrument and its corresponding reagents were purchased from Sebia Company in France.

### 2.4 High‐Performance Liquid Chromatography (HPLC) Analysis

Bio‐Rad Variant II analyser from Bio‐Rad company of the United States was used.

### 2.5 Common Gene Detection of Thalassemia

By using Gap‐PCR to detect the common α‐thalassemia mutations, RDB to detect the common β‐thalassemia mutations with kit from Yaneng Biosciences in China.

### 2.6 DNA Sequencing

The PCR reagents were predominantly supplied by Shanghai Bio‐Work Bioengineering Co., Ltd. and Takara Bio Engineering (Dalian) Co., Ltd., Japan. The returned peak plots and sequences were processed and analysed using biological software such as BioEdit and Chromas and then compared with the normal globin genes in the NCBI database. The sequencing primarily focused on the HBA and HBB genes.

## 3 Results

**FIGURE 1:**
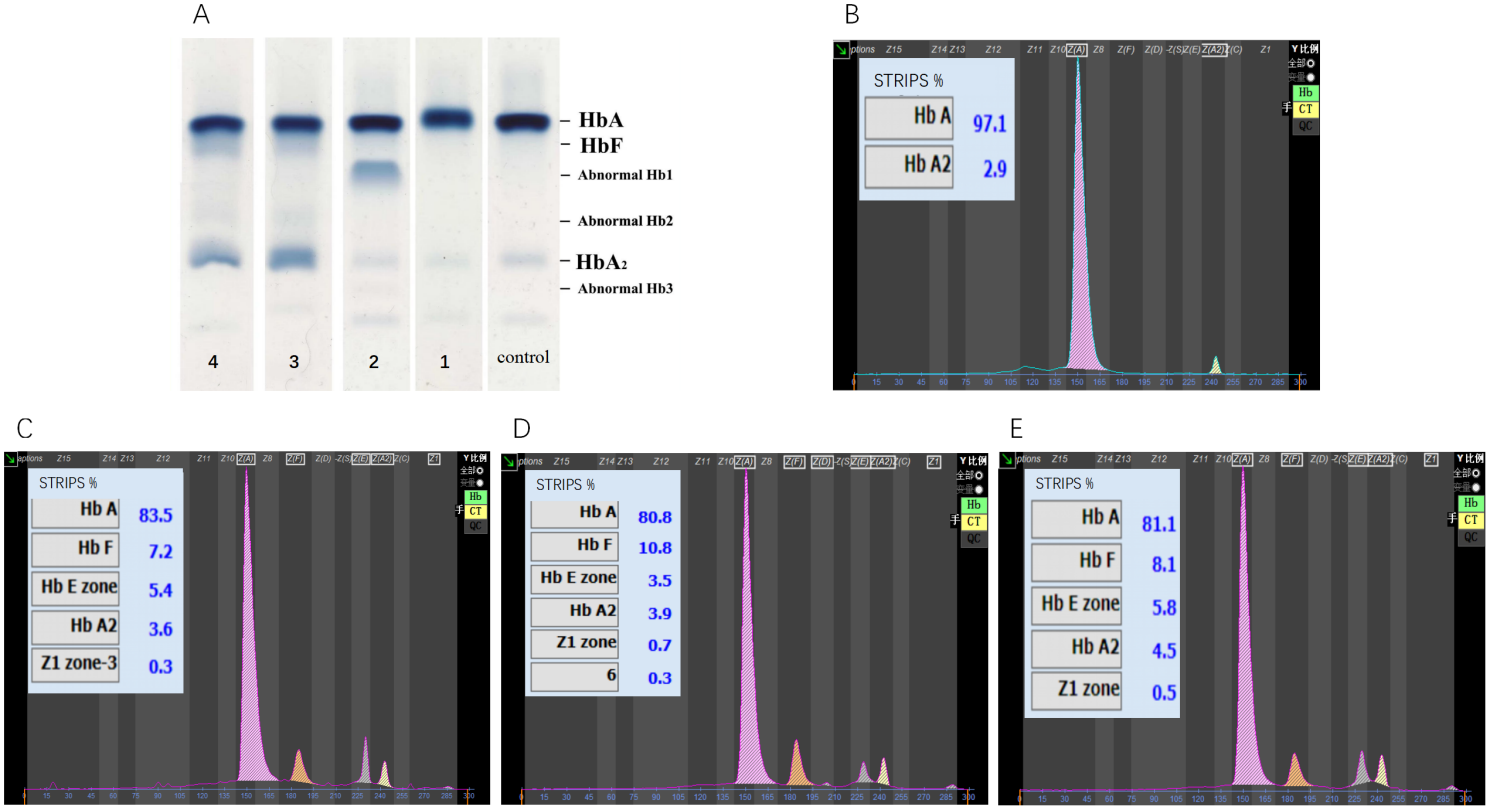
Hb electrophoresis results. (A) pH 8.6 agarose Hb electrophoresis results. Control: Hb electrophoresis map of normal adult; 1–4: Patients 1–4. (B–E) Hb electrophoresis maps of patients 1, 3–5, respectively.

In this study, we found that patient 1 exhibited microcytic hypochromic anaemia; patient 2 had normal Hb levels with only a reduced MCH, presenting a hypochromic anaemic phenotype. Patients 3 and 4 both had macrocytic anaemia, while patient 5 had decreased Hb levels (Table 1). Three types of electrophoretic behaviour were observed by alkaline gel electrophoresis and CE methods. The agarose gel electrophoresis (Figure 1A) showed that only had a shift in the Hb A band, with Hb A_2_ at approximately 2.10%, but no abnormal Hb was observed obviously in patient 1. Patient 2 exhibited two abnormal Hb bands, identified as Hb1 and Hb3, with quantities of about 23.38% and 0.39%, respectively. Patients 3 and 4, a mother‐daughter pair, showed a significant amount of Hb F, a Hb2 band near the position of Hb A_2_ and an abnormal Hb at the Hb A_2_ position. Patient 3 had Hb F, abnormal Hb2 and Hb A_2_ + abnormal Hb amounts of approximately 8.44%, 4.24% and 23.32%, respectively; Patient 4 had corresponding values of about 10.51%, 3.37% and 18.50%. Patient 1 and patients 3–5 were all tested using the CE methods (Figure 1B–E and Table 1).

**TABLE 1:** Complete blood count, Hb electrophoresis, genetic diagnosis results and Hb variants.

Patient	Sex	Age	RBC (×10^12^/L)	Hb (g/L)	MCV (fL)	MCH (pg)	RDW‐CV	Abnormal Hb (%)	Hb (A + F)* HbA/HbF (%)	HbA_2_ (%)	HBA genotype	HBB genotype	Haemoglobin variants	HGVS name	Residue	Substitution
1	M	65	2.98	64	71.5	21.5	0.16	0	97.1/0	2.9	αα/αα	β^Fukuyama^/β	Hb Fukuyama	HBB:c.232 C>T	77 (EF1)	His>Tyr
2	M	31	5.27	139	86.4	26.4	0.14	23.77	73.56*	2.67	α^Port Phillip^α/αα	β/β	Hb Port Phillip	HBA2:c.275T>C	91 (FG3)	Leu>Pro
3	F	23	3.95	116	102.2	29.4	0.16	5.7	83.5/7.2	3.6	αα/αα	β^Saint Etienne^/β	Hb Saint Etienne	HBB:c.279C>G	92 (F8)	His>Gln
4	F	4	4.13	109	94.5	26.4	0.17	4.5	80.8/10.8	3.9	αα/αα	β^Saint Etienne^/β	Hb Saint Etienne	HBB:c.279C>G	92 (F8)	His>Gln
5	F	29	2.75	88	125.1	32	0.15	6.3	81.1/8.1	4.5	αα/αα	β^Saint Etienne^/β	Hb Saint Etienne	HBB:c.279C>G	92 (F8)	His>Gln

Abbreviation: RDW, red blood cell distribution width.

* represents the numerical value of Hb (A+F).

Subsequently, abnormal bands with a content of 39.0% were eluted from patient 1 at 1.812 min, 13.8% and 0.3% were eluted from patient 2 at 3.020 and 3.952 min, and 11.1% were eluted from patient 4 at 4.7 min by HPLC analysis. Meanwhile the results of degenerative globin body and common gene detection of thalassemia in all subjects were normal.

**FIGURE 2:**
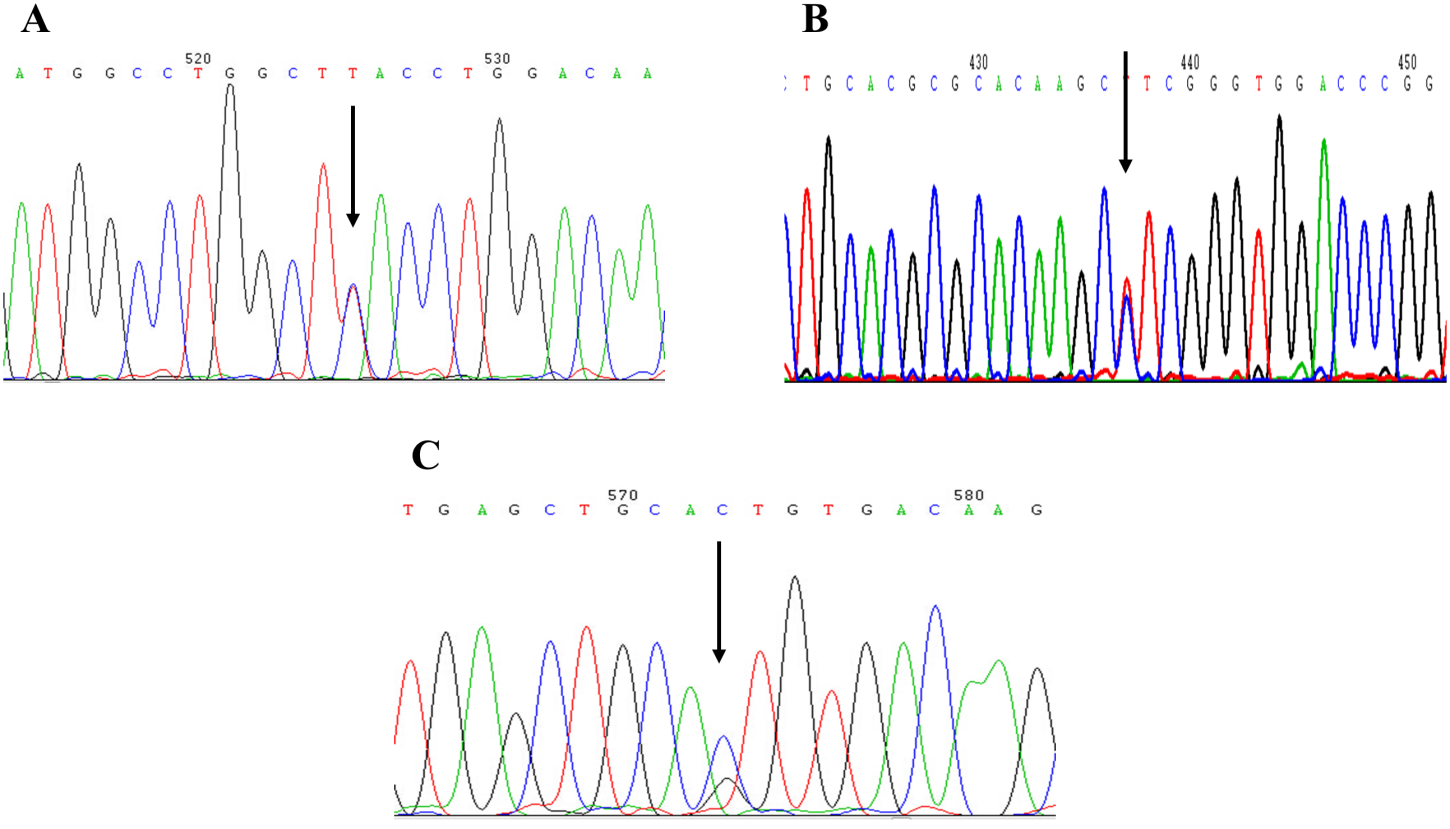
Sequencing results of three kinds of haemoglobin variants. The mutation sites were indicated by arrows. (A) Patient 1 Hb Fukuyama (HBB:c.232C>T); (B) Patient 2 Hb Port Phillip (HBA2:c.275T>C); (C) Patients 3–5 Hb Saint Etienne (HBB:c.279C>G).

Finally, all these five patients of haemoglobin variants were sequenced. The DNA sequencing results of three types of abnormal haemoglobin were shown in Figure 2; Table 1.

## 4 Discussion

Our study identified three rare abnormal Hb species, including Hb Fukuyama (HBB:c.232C>T), Hb Port Phillip (HBA2:c.275T>C) and Hb Saint Etienne (HBB:c.279C>G). Hb Fukuyama (HBB:c.232C>T) has been discovered for the first time in the Chinese population.

Hb Fukuyama is a rare abnormal Hb variant characterized by a very rare substitution at position β77 (EF1) in the globin chain (His>Tyr), resulting from a missense mutation in the β‐globin gene (HBB:c.232C>T). Since this mutation was first observed in Japan by Hidaka et al. (1988), it was subsequently found in the United States, Indonesia and Sweden (Landin and Jeppsson 1993). Our research confirmed the existence of such an abnormal haemoglobin in the Chinese population for the first time, which indicated that Hb Fukuyama (HBB:c.232C>T) also exists in mainland China. Patient 1 presented to the haematology outpatient clinic with moderate anaemia, characterized by microcytic hypochromic anaemia on CBC, and a very low level of ferritin (2.9 ng/mL). Hb electrophoresis performed using the alkaline agarose method revealed a reduction in Hb A_2_, with the Hb A band only slightly shifted upward and no distinct abnormal bands resolved, which consistent with the literature reports (Hidaka et al. 1988; Moo‐Penn et al. 1991). In this study, no abnormal structures were found by CE. According to the guidelines of the British Haematology Standards Committee, different techniques are needed to confirm unknown haemoglobin variants (Bain et al. 2023). In this study, HPLC was employed to analyse haemoglobin, resulting in the elution of an abnormal band with a proportion of 39.0%. Since isoelectric focusing (IEF) was not utilized in this study to analyse this abnormal haemoglobin, a review of the literature (Hidaka et al. 1988) indicates that IEF can resolve such bands, thereby preventing the occurrence of false negatives in potentially heterozygous cases. Its high resolution prevents band overlap (Chalumeau et al. 2021). Therefore, when no distinct abnormal haemoglobin bands are identified by alkaline agarose electrophoresis or CE techniques, HPLC or IEF may be employed for detection. This patient had no history of blood transfusion, hepatosplenomegaly, jaundice, gallstones or chronic haemolysis. After 6 months of iron supplementation, ferritin, MCV and MCH returned to normal levels, with Hb at 104 g/L. Haematological examination was normal, with no signs of haemolysis; the previous moderate anaemia with reduced MCV and MCH was attributed to microcytic hypochromic anaemia caused by iron deficiency. The diagnosis of Hb Fukuyama was confirmed through DNA sequencing. Smit et al. (1991) mentioned in their article a family where the mother had compound α^0^‐thalassaemia (‐‐^SEA^/αα), presenting with mild anaemia characterized by microcytosis and hypochromia typical of α‐thalassemia. This suggests that the β‐variant compound with α^0^‐thalassemia mutations does not mitigate the clinical phenotype of α‐thalassemia. Hidaka et al. (1988) proposed that the Hb Fukuyama mutation involves an amino acid residue at position β77, located within the internal non‐helical segment (EF1) of the β^A^ chain, which does not participate in heme–heme interactions or any inter‐chain contacts within the haemoglobin molecule. Therefore, carriers do not exhibit any haematological or clinical abnormalities. It is clinically benign.

Hb Port Phillip is a rare abnormal Hb variant caused by a missense mutation (HBA2:c.275T>C) on the α2‐globin gene, resulting in a chain substitution (Leu>Pro) at α2 91(FG3). It was first reported in a Chinese female by Brennan, Tauro, and Melrose (1977). Subsequently, Li et al. (2021) identified it in two Chinese families. In our study, Patient 2 was a Chinese male with no apparent clinical symptoms. Routine blood tests showed a Hb level of 139 g/L, normal MCV and only slightly low MCH. The Heinz body test was negative. HPLC analysis of Hb revealed an abnormal band with a proportion of 14.1%. Alkaline agarose gel electrophoresis showed two abnormal Hb bands near Hb A. Sequencing analysis confirmed the diagnosis of Hb Port Phillip. The patient exhibited no anaemic symptoms, similar to the α_2_‐globin gene variant reported by Li et al. (2021) and Yang et al. (2021), which also had no clinical symptoms or haematological impact. This mutation is due to the loss of contact with the haemoglobin molecule and the substitution of proline for leucine. Therefore, Hb Port Phillip is clinically benign similarly.

Hb variants typically lead to structural changes in the globin molecule, affecting the stability of the globin and making it more susceptible to degradation. The phenotypic spectrum of Hb variants can range from asymptomatic (as seen in patients 1 and 2) to severe clinical presentations (Li et al. 2021) (as seen in patients 3–5).

Hb Saint Etienne also known as Hb Istanbul, is a rare unstable abnormal Hb variant. It is caused by a missense mutation (HBB:c.279C>G) on the β‐globin gene, resulting in a chain substitution (His>Gln) at position β92(F8). This is a rare inherited haemoglobinopathy that leads to the loss of heme from the β‐globin chain. The common clinical manifestations include chronic haemolytic anaemia, jaundice, splenomegaly and gallstones. According to the HbVar database (https://globin.bx.psu.edu/globin/hbvar/) and the report by Aksoy et al. (1972), the variant carries heme only on its α‐chain, which affects the quantification of the abnormal haemoglobin. The proportion in heterozygotes is approximately 5%–20%. Compared to many other β‐chain haemoglobin variants, this low percentage may be due to the instability of the abnormal haemoglobin molecule or a reduced rate of synthesis. In this study, the proband (patient 3), her daughter (patient 4) and her sister (patient 5) had abnormal Hb levels of 4.5%–6.3% by CE analysis. CE also showed elevated levels of HbA_2_ at 3.6%–4.5%, a common finding in unstable β‐chain haemoglobinopathies. The former two exhibited relatively small amounts of abnormal Hb near the Hb A_2_ position and a larger amount of abnormal Hb bands at the Hb A_2_ position on alkaline agarose electrophoresis. The daughter's HPLC eluted an abnormal band at 11.1% with a peak time of 4.7 min, consistent with the report by Au et al. (2009). The Hb F levels in the proband, her daughter and her sister ranged from 7.2% to 10.8%, with the daughter having a relatively high level of Hb F at 10.8%, which may correlate with her milder symptoms, a notion supported by literature (Au et al. 2009). Chronic haemolytic anaemia is characterized by an increase in MCV and a rise in reticulocyte count. The MCV for the proband, her sister and her daughter were 102.2, 125.1 and 94.5 fL, respectively, with the proband's reticulocyte count at 0.125%. This is consistent with the report by Jian‐Xin and Gao (2019). All three presented with jaundice and splenomegaly, the proband also had splenomegaly and gallstones, with total bilirubin reaching up to 90.6 μmol/L before splenectomy and cholecystectomy, after which symptoms improved. The clinical symptoms and haematological parameters are in line with those reported for unstable Hb Bushwick, Hb Santa Ana by Kitazawa et al. (2000) and Li et al. (2022). The three patients in this family were ultimately diagnosed with Hb Saint Etienne (HBB:c.279C>G) through sequencing analysis, resolving the aetiology that had puzzled them for years. This abnormal Hb is pathogenic and highly unstable, with the histidine residue near the proximal iron of the heme being replaced, increasing the instability of the heme–globin bond, and even leading to heme/globin dissociation, which significantly disrupts the normal function of the molecule (Aksoy et al. 1972).

In summary, this study identified five cases of abnormal Hb: Hb Fukuyama (HBB:c.232C>T), Hb Port Phillip (HBA2:c.275T>C) and Hb Saint Etienne (HBB:c.279C>G), and analysed their haematological characteristics and clinical features. The combined application of HPLC and CE techniques for the initial screening of abnormal haemoglobin is essential and can reduce the rate of missed diagnoses. Regrettably, not all patients in this study were subjected to both two methods, and it is hoped that it would be addressed in future studies. To sum up, this study has important guiding significance for the screening of abnormal haemoglobin, especially for the epidemiological investigation of abnormal haemoglobin, which is rare and easy to miss. It is of great significance to prevent the birth of children with severe clinical symptoms.

## Author Contributions

Yanfen Ge, Guansheng Zheng and Huizhuang Shan were involved in the design of the study. Yanfen Ge, Junru Liu, Yanfei Luo have developed the research. Luhua Xian, Ting Lin developed the methodology. Wenhao Cui, Yujing Yang were responsible for clinical data collection. Yanfen Ge, Guansheng Zheng were responsible for drafting of manuscript. All authors reviewed and approved the manuscript.

## Ethics Statement

We obtained written informed consent including data and images for publication from the guardian of the patient. This study was approved by the Ethics Committee of Guangdong Provincial People's Hospital (KY‐Q‐2022‐481‐01).

## Conflicts of Interest

The authors declare no conflicts of interest.

## Data Availability

Research data are not shared.
